# Comparative studies need to rely both on sound natural history data and on excellent statistical analysis

**DOI:** 10.1098/rsos.170346

**Published:** 2017-09-20

**Authors:** Carsten Schradin

**Affiliations:** 1 Université de Strasbourg, CNRS, IPHC UMR 7178, 67000 Strasbourg, France; 2 School of Animal, Plant and Environmental Sciences, University of the Witwatersrand, Johannesburg, Wits 2050, South Africa

Evolutionary biologists aim to determine why a particular trait evolved. Detailed studies of single species can identify fitness benefits of specific traits and why those traits evolved, but these results might be rather species specific. Thus, to reach general conclusions about which ecological factors favour the evolution of a specific trait, comparative studies in several taxa are needed. Currently, comparative studies arising from databases summarizing many field studies are flourishing and provide important insights for our understanding of social evolution [1–3]. Large, diverse datasets require detailed specialist knowledge in a wide variety of systems or taxa that is very rare, making it difficult to assess the quality of such databases. Referees of papers submitted to assess these comparative studies should review the part of the database for which they do have specialized knowledge. In some cases, studies with large databases could benefit from one additional referee that focuses only on the database itself, just as the views of specialized referees are often sought on aspects of statistical analysis or of animal ethics. In sum, the quality of a study depends on the quality of the data, and this is also the case for comparative studies.

A recently published comparative study in Royal Society Open Science about cooperative breeding in mammals [4] used a database previously used in a comparative study of the evolution of monogamy in the same taxa [5]. It had already been shown that for the third largest mammalian order, the Eulipotyphla, the information in this database is, to a large extent, inadequate [6]. In the supplement to their earlier publication on the evolution of social monogamy [5], Lukas & Clutton-Brock state that the database is the result of a systemic literature review on ‘mammalian species…collecting information from the primary literature…from encyclopedias and from published reviews'. The reference provided for the Soricomorpha (the mole-shrew-solenodon clade, which is the largest within the Eulipotyphla) is a descriptive report about the presence of the shrew *Sorex alpinus* in the Carpathian Mountains of Romania [7]. It does not provide data on the social organization of this or any other shrew species. Citing this single-species publication, Lukas & Clutton-Brock conclude that all but one of the 367 species of shrews are solitary with the exception of the Etruscan shrew *Suncus etruscus*, which is stated to be socially monogamous. However, this species is not mentioned in the reference provided [7]. While to my knowledge no field studies exist about the social organization of *S. etruscus*, a study published in 1974 in French indicates that this species can be kept in pairs in captivity [8]. Whether this implies that it is socially monogamous in nature is not known.

Lukas & Clutton-Brock categorized 366 of the 367 species of shrews as solitary, based on the statement that ‘The representatives of the Soricidae are solitary’ in [7]. Of the 367 species of Soricidae included in their database, to my knowledge only 11 have been studied in the field during the breeding season regarding their social organization (table 1). To my knowledge, no reliable field data on social organization exists for the remaining 356 species [6] (though the social and mating behaviour of some more species have been studied in the laboratory, or trapping was done in the field without describing the social organization). Of these, four were reported to be solitary, four pair-living and three group-living during the breeding season [6]. While there are statistical methods to neutrally calculate missing independent variables in large datasets (to be able to use the data which are available for other independent variables; for example, environmental variable of a species with known social organization), these methods cannot be applied for the dependent variable (in this case the social organization of a species). Thus, from the data published in the primary literature that 64% of the 11 studied species are pair- or group-living during the breeding season, it is not evident why 99% of all shrew species should be regarded as solitary-living, especially as no data exist for the majority of these species.

**Table 1.** Categorization of the social organization of different species of shrews during the breeding season by Lukas & Clutton-Brock [5], and by Valomy *et al.* ([6] based on primary literature). Lukas & Clutton-Brock define species as monogamous only if pairs stay together for more than 1 year, while Valomy *et al*. also consider a male and a female that stay together during only one breeding season as a pair (which was categorized as solitary by Lukas & Clutton-Brock).

species	origins	Lukas & Clutton-Brock	Valomy *et al*.	agreement?
*Crocidura leucodon*	Europe	solitary	pair	no
*Crocidura russula*	Europe	solitary	pair	no
*Crocidura shantungensis*	China	solitary	solitary	yes
*Cryptotis parva*	North America	solitary	group	no
*Neomys fodiens*	Eurasia	solitary	solitary	yes
*Sorex araneus*	Eurasia	solitary	solitary	yes
*Sorex cinereus*	North America	solitary	group	no
*Sorex coronatus*	Europe	solitary	pair	no
*Sorex ornatus*	North America	solitary	group	no
*Sorex unguiculatus*	Russia and China	solitary	solitary	yes
*Suncus varilla*	South Africa	solitary	pair	no
*Suncus etruscus*	Europe	monogamous	no primary information found	no
355 other Soricidae species		solitary	no primary information found	no

The reason for the discrepancy between the two studies in categorizing might be differences in the definitions used. Though both studies emphasize that their classification focuses on social organization (i.e. group composition [9]), Lukas & Clutton-Brock also focus on the breeding strategy of females. Valomy *et al*. [6] categorized all species where adults did not only meet for mating but also lived in pairs during the breeding season as pair-living, independent of the duration for which pairs stayed together. Lukas & Clutton-Brock categorized species as monogamous or solitary breeding (and as group-living if two or more breeding females shared a home range), indicating that maybe they did not only consider the social organization, but also the mating system. They classified species as monogamous when adult pairs shared a home range for at least 1 year (electronic supplement of [5]; more than 50% of breeding females had to be pair-living for more than 1 year). Thus, pairs that did not live for more than 1 year were not categorized as monogamous. As Eulipotyphla typically do not live for more than 1 year as adults [10,11], this specific definition could explain why most of them were not regarded as monogamous, but as solitary. In sum, whereas Valomy *et al*. classified shrews as pair-living when one male and one female stayed together for longer than only mating during the breeding season, sharing a home range and nest with young, Lukas & Clutton-Brock classified these as solitary as these pairs did not stay together for more than 1 year, and females of these species were considered as solitary breeders in their database.

Though these differences in definition can explain the discrepancies in categorizing the species that have been studied in the field (table 1), several problems remain. First, if an adult average life-expectancy of less than 1 year excludes for these species (by definition) another form of social categorization than ‘solitary’, then ‘solitary’ has a different meaning in such a subset than for other, longer-lived species. In that case, short-lived species like shrews would create structural zeros: structural missing categories in these species means that the cell cannot vary. Inferences would therefore be invalid, for biased parameter estimates, as well as incoherent meaning of the social categories [12]. Thus, these species would have to be excluded from the database, because they cannot be reliably categorized. Second, Lukas & Clutton-Brock categorize one of the smallest mammals, the Etruscan shrew *Suncus etruscus*, as monogamous. While under artificial conditions in captivity this species can live for more than 3 years, both in the field and in captivity this species breeds only for one breeding season of six (field) or 7.5 (captivity) months [11]. Thus, considering their definition, they wrongly classified the Etruscan shrew as monogamous. Third, Lukas & Clutton-Brock categorize several species of the genera *Microtus* and *Peromyscus* as socially monogamous. With this they follow the classical categorization for these species [13,14], but not their own classification. It is very unlikely that in these species more than 50% of pairs live together for more than 1 year under natural conditions, as for these rodents average adult lifespan in nature is typically much less than 1 year [15,16] (and for their definition both pair partners would have to survive for more than 1 year). In fact, ‘virtually no animal (=vole, inserted by CS) that breeds in one year is alive the next’ year [15]. For example, in the socially monogamous prairie vole *Microtus ochrogaster*, mean survival rate is only 65 days [17] and adult individuals survive only for approximately eight weeks after their first capture [18]. Thus, their database includes wrongly classified species (if one uses their own definition) and many species which because of their short life-expectancy cannot be reliably classified using their definition.

In comparative studies, the quality of the database might be poor if researchers focus on obtaining information from as many species as possible (a ‘complete’ database), instead of sourcing the most reliable information, which would lead to a much smaller database. Fifty years ago, we had nearly no information on the social organization of mammals under natural conditions. Though many species have now been studied [19], information on the large majority is still missing. If we do not have sufficient reliable data for comparative studies at the moment, then we might have to wait until such data have accumulated. Even for some of the 11 shrew species which have been studied in the field (table 1), the quality of the data might be low and it might be difficult to correctly categorize their social organization. In this case, these species should be excluded from comparative studies until reliable data become available.

To obtain a complete database, assumptions based on phylogenetic relatedness enter such databases. For example, in a comparative study about parental care in birds, if all members of a genus had the same reported mode of parental care, the same mode was assumed for species of the same genus which had not been studied [20]. Similarly, the Lukas & Clutton-Brock database contains entries for hundreds of species for which no information about their social organization is available from field studies, based on studies on a few closely related species. Their hypotheses are important and sensible; my criticism does not make a statement whether or not these hypotheses are correct. However, I do recommend that comparative studies should make no assumptions of species not studied, even when phylogenetically related species have been studied in detail. Instead, species with no data should be excluded from databases.

In the last few decades, the focus in animal behaviour has been theory-based research, with little attention and funding for descriptive studies. However, the quality of comparative studies not only depends on R programming abilities, but also on the quality of the data used. To obtain good comparative databases for theory-driven research, we need high-quality field studies, including studies describing the social system (including the social organization [9]) of species. There have been requests to increase efforts and funding for describing the world's biodiversity taxonomically [21]. Similarly, we should also increase our efforts to describe the biodiversity of social systems if we believe social evolution is an important topic of research. In summary, to study social evolution, comparative studies must be based on reliable information and this requires many more species to be studied carefully in their natural environment.

## Data accessibility

Data on social organization of Eulipotyphla are available as electronic supplement of Valomy *et al*. [6].
